# Positive Self-Perceptions of Aging Play a Significant Role in Predicting Physical Performance among Community-Dwelling Older Adults

**DOI:** 10.3390/ijerph182111151

**Published:** 2021-10-23

**Authors:** Emma Nilsson, Helena Igelström, Irene Vikman, Agneta Larsson, Mascha Pauelsen

**Affiliations:** 1 Department of Health, Education and Technology, Luleå University of Technology, SE 971 87 Luleå, Sweden; ehn91_@hotmail.com (E.N.); irene.vikman@ltu.se (I.V.); agneta.larsson@ltu.se (A.L.); 2 Department of Neuroscience, Physiotherapy, Uppsala University, SE 752 37 Uppsala, Sweden; helena.igelstrom@neuro.uu.se

**Keywords:** self-perceptions of aging, physical functional performance, attitude toward own aging, ageism, falls, healthy aging

## Abstract

Self-perceptions of aging (SPA) is associated with various health-related outcomes, including physical performance. No previous study has investigated the potential predictive influence of SPA on physical performance among Swedish community-dwelling older adults. This was a cross-sectional study using a random sample of 153 Swedish community-dwelling individuals aged 70 and older. Multiple logistic regression analysis was performed, using the subscale “Attitude Towards Own Aging” of the Philadelphia Geriatric Center Morale Scale, as a measure of SPA. The Short Physical Performance Battery (SPPB) was dichotomized and used as the outcome variable. SPA was a significant predictor (OR = 1.546, CI = 1.066–2.243) of physical performance, adjusted for age, cognitive function, and life-space mobility. Further analyses revealed significant sex differences, with SPA not being included in the model for the men whilst it was still a significant predictor (OR = 1.689, CI = 1.031–2.765) of physical performance in the group of women. SPA plays a significant role in predicting physical performance among Swedish community-dwelling older adults. To further clarify this relationship and its consequences, future longitudinal research should focus on the relationship between SPA, physical performance, and fall risk.

## 1. Introduction

Worldwide, the proportion of older individuals is increasing faster than any other age group [1]. Sweden is no exception, as approximately 15% of the total population is constituted by individuals aged 70 and older [2] and is projected to be double that by 2050 [1]. This increase leads to new opportunities and challenges, such as achieving healthy aging. To do so, maintaining functional ability such as physical performance is of great importance [3]. Physical performance is regulated by factors associated with nervous, skeletal, and muscular systems, which can be influenced by biological and psychosocial factors as well as lifestyle [4]. Physically, the process of aging is associated with a decline in muscle mass which can lead to diminished musculoskeletal function and strength. Age-related decline in physical functions such as gait speed, leg strength, and balance have been associated with fall prevalence and reoccurrence [5] as well as with fall-related concern [6,7]. In addition, factors such as older age and physical performance also relate to daily mobility within and between different geographical areas in the community [8]. It is of importance though to highlight that the impact of age-related changes is diverse across individuals and physical limitations due to aging can be managed by engaging in physical activity or exercise [9,10].

How older individuals perceive their age and aging is associated with various health-related outcomes including physical performance [11,12]. To maintain healthy aging and limit the impact of declined physical performance, it is of importance to increase the understanding of factors related to physical performance, such as self-perceptions of aging (SPA). Beliefs and expectations of aging are hypothesized to assimilate during childhood, reinforced during the lifespan and in older adulthood develop into SPA [13]. The mechanisms by which SPA affects health are not completely clarified, but SPA is thought to affect health through behavioral, physiological, and psychological pathways [14,15,16,17].

When we, explicitly or implicitly, let chronological age shape how we think of, feel about, or act on others and ourselves, it is called ageism [18]. Ageism, and negative SPA as a form of ageism, is highly prevalent all over the world [19], adding to the individual and societal cost of an aging population both healthwise and economically [20]. Previous longitudinal research investigating the direction of the relationship between SPA and physical functioning indicate SPA to affect physical functioning and not vice versa [14,21,22]. Longitudinal studies have also shown both positive and negative SPA to be associated with physical performance [21,22,23]. However, due to the studied population in those studies, the results are difficult to generalize to a Swedish population of independent adults aged 70 and older.

The specific relationship between SPA and physical functioning has been established by several authors [21,22,23]. The number of studies exploring this relationship using objective measures of physical performance is limited and to our knowledge, no such previous study exists based on a Swedish population. The purpose of this study is to investigate if self-perceptions of aging can act as an independent predictor of physical performance, among Swedish community-dwelling older adults.

## 2. Materials and Methods

The data in this study originates from a previously published study by Pauelsen et al. [6], which aimed to describe the prevalence of fall-related concern and find explanatory factors of falls self-efficacy. Data were collected during home visits, performed by either one of four licensed physiotherapists (authors IV, MP, and two others).

### 2.1. Population and Sampling

A random population sample of 362 people aged 70 and over were contacted by telephone to participate in the main study. Of those, 153 (42%) agreed to participate. Inclusion criteria were community living residents in a specific northern municipality in Sweden, aged 70 years or older. People who could not make or keep the visit appointments themselves due to cognitive ability, were excluded. See Pauelsen et al. [6] for a more detailed description of the process.

### 2.2. Measures and Data Preparation

The “Attitude Toward Own Aging” (ATOA) subscale of the Philadelphia Geriatric Center Morale Scale (PGCMS) was used as a measure of self-perceptions of aging, reflecting self-directed ageism. We used the Swedish-translated British-English version [24] of the PGCMS [25], in which the ATOA subscale is made up of 5 yes/no items. The ATOA subscale asks the participants to rate their own aging experience and compare themselves now to a younger self. An example item is “Are you as happy now as you were when you were young?” [24]. The more positive answer at each question marks a point, resulting in a total score of 0–5 points. The sum score of the subscale ATOA was used in the analyses. This subscale is regularly used as a measure of self-perceptions of aging, has demonstrated good reliability (Cronbach’s alpha 0.62–0.82) [12] and as all five items load highly on a single factor the ATOA subscale has good construct validity [22].

To assess physical functional performance the Short Physical Performance Battery (SPPB) was used, which consists of functional tests including standing balance, walking, and chair stands [26]. Total scores range from 0 to 12, with higher scores indicating better physical function. The sum score was used in the correlations but was dichotomized for the logistic regression: low SPPB (0–10 points) and high SPPB (11–12 points).

The Mini-Mental State Examination (MMSE) was used as a brief screening test of cognitive impairment with scores ranging from 0 to 30, with a higher score indicating less cognitive decline [27]. As a measure of mobility, the University of Alabama at Birmingham Life-Space Assessment (LSA) was used by structured face-to-face interviews [28]. The measure requires the participants to recall how often and independently (i.e., need for mobility aid/assistance or not) they have purposely moved through different spatial areas during four weeks prior to the assessment. The composite score can range from 0 (totally confined to bed) to 120 (independent, with daily out-of-town mobility). The sum score of MMSE and LSA, respectively, were used in the analyses.

Self-rated health was assessed by asking the participants the following “Compared to other people your age, do you perceive your health status worse than others, same as others, better than others or do not know?”. The variable was dichotomized to “Same as others” and “Better than others” as one category, and “Worse than others” as a reference category. Answers “Do not know” were considered as missing data.

To assess fall history, participants were asked how many times they had fallen during the last 6 months, for which the definition of a fall was: “an unexpected event in which the participant comes to rest on the ground, floor, or lower level” [29]. In the analyses this variable was dichotomized to “No previous falls” in one category and “One or more previous falls” used as a reference category. The Swedish version of Falls Efficacy Scale-International (FES-I(S)) was used to measure how concerned the participant was about falling whilst doing certain activities [30], with a total sum-score of 16–64 where a higher score indicates a lower falls self-efficacy. To assess fear of falling the participants were asked a single-item question “Are you afraid of falling?” answered on a Likert scale from 1 (no, not afraid) to 4 (yes, very much). To assess concerns about consequences of falling the participants were asked four questions, which reflect on some of the most common beliefs concerning the negative consequences of falling [31]. These were also scored on a Likert scale of 1 (no concern) to 4 (very concerned) and were then combined into one variable (Any_CC) to describe the prevalence of any kind of concern about consequences of falling. Both the fear of falling variable and the Any_CC variable were dichotomized with answers including value 1 being the response category (no concern/not afraid) and answers including values 2–4 combined to a reference category (concerned/afraid).

### 2.3. Data Analysis

Descriptive statistics are presented for the whole sample as well as for men and women separately. Nominal variables are presented in frequency and percentage. The residual variables are presented with mean and standard deviation as they were treated on an interval/ratio level in the analyses [32]. For comparisons between women and men the *X*^2^, the independent samples t-test, and the Mann–Whitney test was used, where the type of data dictated the choice of test.

To examine if self-perceptions of aging could be a predictor of physical performance when adjusted for covariates, a multiple logistic regression analysis was performed using the dichotomized SPPB variable as the dependent variable with the group of higher values (11–12) used as the response category and the group of lower values (0–10) used as a reference category. The cut-off point for the two subgroups was chosen considering previous studies using the SPPB scale and a concern for small group sizes [5,33,34]. The logistic regression analysis was performed with all control variables entered with the forward stepwise (likelihood ratio) method. To control for the results of the previous analysis it was repeated using the backward stepwise (likelihood ratio) method. Covariates included in the modeling process were ATOA, age, sex, number of medications, previous falls, self-rated health, MMSE, shared living, fear of falling, any consequence concerns (Any_CC), FES-I, ATOA x Sex (interaction variable), home help. In all logistic regression analyses the setting used for entry of variables was *p* ≤ 0.05 and removal of variables was *p* ≥ 0.10.

Due to various SPPB cut-off scores available in the literature, additional logistic regression analyses were performed with the various reported cut-offs as dichotomization guides. Regressions were calculated in which the dependent variable (SPPB) was dichotomized at 7, 8, 9, and 10, respectively. The models in this sensitivity analysis (not presented) showed the same regressors as significant predictors as the main model presented in the results.

Data analyses were performed using SPSS (IBM SPSS Statistics 26, IBM corp., Armonk, NY, USA). Missing data were not imputed.

## 3. Results

The characteristics of the sample are presented in Table 1. Almost 95% of the total sample reported their health to be as good as or better than other people their age. Several variables showed significant sex differences (see Table 1).

There were missing data for 10 participants (8 women, 2 men). The data missing was from the following variables (with the amount of missing data presented within brackets): self-rated health (8), falls past six months (1), number of prescription medications (1), concerns about needing (more) help after a fall (1). Two participants had answered “Do not know” on the question regarding self-rated health, which also was considered as missing data.

### 3.1. Associations to Physical Performance

The logistic regression analysis showed that LSA, age, MMSE, and ATOA were significant variables to predict whether a participant would receive a high SPPB score or not (see model 4, Table 2). Following MMSE, ATOA was the second strongest predictor of high SPPB scores. For every one unit increase on ATOA the odds of receiving high SPPB was almost 55% higher. The classification table showed that the model predicted 86.1% of the responders and 76.1% of the non-responders correctly, adding together to an overall prediction accuracy of 81.5% (data not presented). As can be seen in Table 2, all predictor variables were significant using the Wald statistic, indicating the variables to be significant contributors to the model [35].

### 3.2. Analyses of Gender Differences

Correlational analysis between ATOA and SPPB divided by sex revealed significant differences, with a positive significant correlation found in the group of women (r_s_ = 0.392) whilst no such significant correlation could be found in the group of men (r_s_ = 0.164). Based on this, further logistic regression analyses were performed with models separated by sex. Of all the variables included in the modeling process, LSA (OR = 1.072, CI = 1.034–1.112), MMSE (OR = 1.495, CI = 1.044–2.141), ATOA (OR = 1.689, CI = 1.031–2.765), and shared living (OR = 4.482, CI = 1.274–15.771) were found to be significant contributors/predictors in the group of women. In the group of men, age (OR = 0.771, CI = 0.665–0.894) and MMSE (OR = 2.380, CI = 1.271–4.458) were the only variables found to be significant contributors to the model of all the independent variables included in the modeling process.

## 4. Discussion

The aim of this study was to investigate if self-perceptions of aging (SPA) could act as an independent predictor of objectively measured physical performance among Swedish community-dwelling older adults. We found that SPA plays a significant role in predicting physical performance, which is in line with previous research [14,21,22,23]. The current study supports previous notions about the possible protective impact of positive SPA on physical performance [14,21,22] and extends it to the Swedish population.

SPA have been linked to several different aspects of physical health, including changes in gait and physical performance [11,21,23], which in turn also have been found as major risk factors for falls among older adults [36]. Adding to this, physical performance among older adults has also been associated with fall-related concern [6], which in turn can be linked to the risk of falling [37,38]. Decline in physical performance can thus lead to a double threat when it comes to the risk of falling. A risk which SPA, based on the results from this study, might have an important role in through its strong predictive value on physical performance. Both fall-related concern and falls are highly occurring matters amongst older people worldwide [6,37,39,40], with common consequences that can have serious impact on health and quality of life [37,39,41]. To date, only two studies have investigated the predictive value of SPA regarding falls, with one study presenting inconclusive results [42] whilst the results from the other study imply positive SPA to have a potential protective mechanism against falls [43]. Thus, promoting positive SPA could possibly be of great benefit in prevention of falls. The potential relationship between SPA and fall-related concern has not yet been investigated. Based on the possible impact of SPA on fall-related concern and falls, the vast consequences following these issues and the limited research on this topic, it would be of benefit to further investigate this possible association.

MMSE was the independent variable with the highest odds ratio in the logistic regression analysis. Cognitive and physical function, including physical performance, among older adults have been associated in several studies and seems to have a bidirectional relationship, with the strength of that relationship partly depending on the measures used [44,45]. This relationship has been shown to be robust independent of age, sex, race, and heterogenous health conditions, though with variations among subgroups of people [45].

The results imply increasing chronological age to be associated with decreased odds of receiving high (11–12) SPPB. Aging is associated with physiological changes such as declined muscle mass and muscle strength, which are strong contributors to declined physical performance among older adults [4]. Age is thus an obvious variable to be included in a model predicting physical performance in an aging population. It is, however, of importance to bear in mind that there is great variability in old age and that there is no such thing as a “typical” older adult [3,46]. As physiological age-related changes can be affected and eased by factors such as physical activity and exercise [9,10], comorbidities and functional decline may thus be a measure of biological age rather than a function of chronological age. This is of importance as what we ascribe the variable age also reflects on our own view of aging.

The odds ratio of LSA was very low, which could be due to LSA and SPPB measuring similar but distinct aspects of physical health. LSA is a measure of individual mobility in different spatial areas and the frequency and independence during such movement [28]. LSA is thus no measure of physical activity per se but can perhaps give an indication of a person’s level of physical activity. Based on that notion it is perhaps no surprise that, additionally to age, LSA also was a stable predictor of physical performance in the present study. No previous study has been found investigating life-space mobility as an independent variable explaining the variance in physical performance. Though, as life-space mobility reflects actual community mobility and participation in civic life, there are also cognitive, psychosocial, and environmental components including transportation to consider in the construct of LSA [47]. In addition to level of physical mobility, higher sum scores on LSA can also indicate that a person has a well-functioning and attractive social and physical environment [48] which acts as a supporting and motivating factor to stay physically active.

Worthy of special note were the gender differences found in the logistic regression models separated by sex. The model for women was like the model for the total sample, with the additions of shared living and age. The model for men on the other hand, only showed age and MMSE as significant predictors. Reasons for this finding can only be speculated about, but perhaps stereotypical gender roles (the emotional feminine and the tough masculine [49]) may play a part in it. Previous studies investigating the relationship between SPA and physical performance have controlled for gender in their analyses, but do not discuss the possible impact of gender on the results [21,22,23]. The World Health Organization has reported the relation between SPA and gender to be inconsistent [50]. Based on the results from this study and the limited discussion of the impact of and possible differences between gender in the analyses of SPA and physical performance in previous studies, we would like to suggest future research to further investigate this topic. As SPA is a modifiable factor [51] the gender differences found in this study could be of importance to consider in future research when researching the efficacy of interventions to improve physical performance through modifying SPA.

### Strengths and Limitations

Much of the research on SPA has been conducted in a limited number of countries [11,12]. With SPA being strongly influenced by cultural and regional effects [52,53], this increases the risk of location bias. Quantitative studies regarding SPA in a Swedish population are very limited [12,54] and so far, none has investigated the relationship between SPA and physical performance. This study fills that gap.

Most of the covariates included in this study were skewed, which could limit the possibility to extend the interpretation of the results beyond the current population. Flooring/ceiling effects were most likely due to the age and independence criteria that were set. While it will be difficult to generalize the results to groups outside our target population (younger or living in residential care), there were no significant differences in basic characteristics between our sample and the national population, according to data available at Statistics Sweden (SCB) [2]. In comparison with a Dutch population study [40], the sample used in this study had a similar distribution by sex, self-rated health, previous falls, and living situation. However, the SPPB mean of the current study was somewhat higher than that of a Spanish study [55], indicating a slightly more fit sample.

The decision to use logistic regression techniques was based upon the formation of the data. The control variables included in the logistic regression analysis were mainly based on previous research [14,21,22,23,42] and partly in an exploratory manner. In this study sex, self-rated health, and previous fall(-s) were not significantly correlated with SPPB in the bivariate correlation analyses preceding the regression analyses (not presented). These variables were chosen to still be included in the modeling process since they were thought to be potential clinically important variables [56]. Physical performance is complex and driven by many factors [4]. We aimed to maintain this complexity in our statistical analyses by including the potential regressors presented in the literature and modeling them as possible covariates. For the same reasons, we chose to apply a stepwise method; to explore the many possible variables and their combinations. The method has been criticized for taking important methodological decisions out of the hands of the researcher, whilst it also has been defended as a useful tool for exploratory research [35,56]. The stepwise entry procedure was chosen to obtain the most effective set of variables in predicting the outcome variable. These choices have allowed us to present a strong predictive model of physical performance including biological, psychological, and social factors. Supporting this, cognitive function has a well-established relationship with physical performance [44,45] and the expression of that relationship in our model further adds to the model’s validity.

Five cases were detected with residuals greater than 2, which indicates that they are outliers. Outliers were not removed from the model as that could result in missing important information. As the outliers were such a small part (3,5%) of the total sample the risk of them distorting the logistic regression model was deemed to be low [35]. A total of 10 participants had missing data. With a mean age of 83 ± 5.2 years, these 10 participants were significantly older than the remaining sample but based on the small amount of missing data and that it was from different variables the missing data is not thought to have reduced the representativeness of the sample.

SPPB was in this study divided into a binary variable and by changing an ordinal or numerical variable into a dichotomous variable there is a risk of losing important information [35]. Other authors [5,33] have used different definitions for response and reference categories of SPPB compared to the categories used in the primary analyses of this article. To account for potentially lost information, sensitivity analyses were performed to see if and how the cut-off value of the dichotomization would affect the results. The different cut-off values used in our study did not change the results.

A cross-sectional design was used, which gives information about a population studied at a single point in time and therefore no cause or effect can be determined [32]. As previous research [57] has found SPA to vary across the lifespan it might be preferable, if possible, to use a longitudinal design or to stratify a cross-sectional sample by age to capture the variations of SPA through life.

## 5. Conclusions

Self-perceptions of aging (SPA) is a predictor of physical performance among Swedish community-dwelling individuals 70 years and older, when adjusted for age, cognitive function, and life-space mobility. To further clarify this relationship and its consequences, future longitudinal research should focus on the relationship between SPA, physical performance, and fall risk. Additionally, with SPA being a modifiable predictor of physical performance, the possible gender differences discovered in this study should be investigated with a bigger sample.

## Figures and Tables

**Table 1:** Descriptive information.

	Total	Women	Men
**Characteristics**			
Participants	153	96 (63)	57 (37)
Age, mean ± SD	78.2 ± 6.2	78.6 ± 6.3	77.5 ± 6.1
Shared living, *n* (%)	89 (58)	49 (51)	40 (70) *
Has home help services, *n* (%)	13 (9)	9 (9)	4 (7)
Number of prescription medications, mean ± SD	3.4 ± 2.7	3.6 ± 2.8	3.2 ± 2.6
≥1 fall past 6 months, *n* (%)	45 (30)	23 (24)	22 (39) *
**Clinical assessment measures**			
Self-rated health (better than others), *n* (%)	90 (62)	51 (57)	39 (70)
(as good as others), *n* (%)	47 (32)	33 (37)	14 (25)
(worse than others), *n* (%)	8 (6)	5 (6)	3 (5)
Life space (0–120), mean ± SD	68.6 ± 22.8	63.3 ± 22.8	77.6 ± 20.0 ***
Mini-mental test (0–30), mean ± SD	28.0 ± 2.0	27.8 ± 2.0	28.2 ± 1.8
Short physical performance battery (0–12), mean ± SD	9.4 ± 2.8	9.2 ± 2.9	9.8 ± 2.6
Attitude toward own aging (0–5), mean ± SD	2.9 ± 1.5	2.7 ± 1.5	3.3 ± 1.3 *
Fear of falling, *n* (%)	58 (38)	48 (50.0)	10 (17.5) ***
FES-I (16–64), mean ± SD	22 ± 5.8	28 ± 5.1	20 ± 5.8 **
Reporting any concern about consequences (Any_CC), *n* (%)	90 (59.2)	67 (70.5)	23 (40.4) ***

Note: Data is presented with n (%) or mean ± SD. ¤ = Missing values; number of medications: 1, self-rated health: 8. Abbreviations in the table: FES-I, Falls Efficacy Scale-International. For comparison between women and men the X2 test was used for nominal variables, the Mann–Whitney test was used for ordinal and skewed data and the independent samples *t*-test was used for continues variables. * *p* ≤ 0.05. ** *p* ≤ 0.01. *** *p* = 0.001.

**Table 2:** Logistic regression model, using forward (likelihood ratio) stepwise entry method.

Predictor	Model #1	Model #2	Model #3	Model #4
Variables	OR (95%CI)	OR (95%CI)	OR (95%CI)	OR (95%CI)
LSA	1.077 (1.050–2.248) *	1.056 (1.028–1.085) *	1.054 (1.026–1.084) *	1.042 (1.012–1.071) *
Age		0.854 (0.780–0.935) *	0.856 (0.782–0.938) *	0.837 (0.761–0.921) *
MMSE			1.606 (1.177–2.192) *	1.602 (1.159–2.215) *
ATOA				1.546 (1.066–2.243) *
Constant	−5.179	8.359	−5.096	−3.174
Nagelkerke R^2^	0.422	0.504	0.564	0.594
Hosmer and lemeshow				0.257

Note: Dependent variable SPPB: Low SPPB = 0 (n = 71); High SPPB = 1 (n = 72). Cases included: 143. Abbreviations in the table: LSA, Life space assessment questionnaire; MMSE, Mini mental state exam; ATOA, Attitude toward own aging. Variables included in the modeling process: ATOA, age, sex, number of medications, previous falls, self-rated health, MMSE, shared living, fear of falling, any consequence concerns (Any_CC), FES-I, ATOA x Sex (interaction variable), home help. * *p* ≤ 0.01.

## Data Availability

Data cannot be shared publicly because it contains sensitive data including health status, anthropometrics, age, and location (recruited from a relatively small community). Data are available from the LTU Institutional Data Access Offices: Johan Lundberg Karlsson (contact via dataskydd@ltu.se) for researchers who meet the criteria for access to confidential data.
